# On spatial conditional extremes for ocean storm severity

**DOI:** 10.1002/env.2562

**Published:** 2019-02-26

**Authors:** R. Shooter, E. Ross, J. Tawn, P. Jonathan

**Affiliations:** ^1^ STOR‐i Centre for Doctoral Training, Department of Mathematics and Statistics Lancaster University Lancaster UK; ^2^ Shell Global Solutions International BV Amsterdam The Netherlands; ^3^ Department of Mathematics and Statistics Lancaster University Lancaster UK; ^4^ Shell Research Ltd. London UK

**Keywords:** conditional extremes, nonstationary, significant wave height, spatial dependence

## Abstract

We describe a model for the conditional dependence of a spatial process measured at one or more remote locations given extreme values of the process at a conditioning location, motivated by the conditional extremes methodology of Heffernan and Tawn. Compared to alternative descriptions in terms of max‐stable spatial processes, the model is advantageous because it is conceptually straightforward and admits different forms of extremal dependence (including asymptotic dependence and asymptotic independence). We use the model within a Bayesian framework to estimate the extremal dependence of ocean storm severity (quantified using significant wave height, *H*_*S*_) for locations on spatial transects with approximate east–west (E‐W) and north–south (N‐S) orientations in the northern North Sea (NNS) and central North Sea (CNS). For *H*_*S*_ on the standard Laplace marginal scale, the conditional extremes “linear slope” parameter *α* decays approximately exponentially with distance for all transects. Furthermore, the decay of mean dependence with distance is found to be faster in CNS than NNS. The persistence of mean dependence is greatest for the E‐W transect in NNS, potentially because this transect is approximately aligned with the direction of propagation of the most severe storms in the region.

## 1 INTRODUCTION

Quantifying extreme ocean environments is important for safe and reliable construction and operation of offshore and coastal infrastructure. Extreme value analysis provides a framework within which the marginal and dependence characteristics of extreme ocean environments can be estimated and joint inferences corresponding to very long periods of observation made in the presence of nonstationarity with respect to covariates.

The spatial structure of ocean surface roughness within a storm is of particular concern when inferences are based on observations from multiple locations in a neighbourhood. For a given ocean basin, when the distance between two locations is small relative to the spatial extent of a storm low pressure field, it is reasonable to expect that large values of ocean surface roughness (for a period of time of the order of an hour, quantified in terms of significant wave height *H*_*S*_) at the two locations will be dependent. Moreover, the extent of this spatial dependence will potentially itself be nonstationary with respect to covariates, such as storm direction and season. A reasonable statistical description of *H*_*S*_ on a neighbourhood of locations should therefore admit appropriately flexible descriptions of extremal spatial dependence. Incorrect specification or estimation of the dependence structure can lead to misleading joint predictions of *H*_*S*_ on the neighbourhood. We note a number of recent articles on spatial extremes with at least some synoptic content, including Davison, Padoan, and Ribatet (2012), Reich and Shaby (2012), Ribatet (2013), Huser and Wadsworth (2018), and Tawn, Shooter, Towe, and Lamb (2018).

A number of recent studies explore the extremal spatial dependence of *H*_*S*_. For example, Kereszturi, Tawn, and Jonathan (2016) assess the extremal dependence of North Sea storm severity using the summary statistics *χ* and $\bar{\chi }$ (or equivalently, *η*; Coles, Heffernan, & Tawn, 1999), outlined in Section 3. Estimates for these summary statistics were used to categorise observed extremal dependence as either asymptotic dependence (AD; suggesting that extreme events tend to occur simultaneously) or asymptotic independence (AI; suggesting that extreme events are unlikely to occur together); further discussion of these concepts is given in Section 3.1. In Kereszturi et al. (2016), it was found that, in most cases considered, AI seemed to be the more appropriate assumption, compared to the assumption of AD. Kereszturi (2017) and Ross, Kereszturi, van Nee, Randell, and Jonathan (2017) extend this assessment to include the estimation of a number of max‐stable process (MSP) and inverted MSP models (Wadsworth & Tawn, 2012), including the so‐called Smith (Smith, 1990), Schlather (Schlather, 2002), and Brown–Resnick (Brown & Resnick, 1977) models, and corresponding models for the inverted processes. For all models considered, there is evidence that the extremal dependence of *H*_*S*_ at two locations varies with the distance between the locations and their relative orientation.

By construction, MSP models considered in Kereszturi (2017) and Ross et al. (2017) exhibit AD exclusively, whereas inverted MSP models only exhibit AI. In general, we do not know a priori which form of extremal dependence is more appropriate. A decision concerning the form of extremal dependence present in the sample must therefore be made before parameter estimation; this is less than ideal, although estimation of *χ* and $\bar{\chi }$ can aid this choice. We note alternative AD models including those of Reich and Shaby (2012); Ferreira and de Haan (2014); Rootzén, Segers, and Wadsworth (2018); Kiriliouk, Rootzén, Segers, and Wadsworth (2018). A number of more sophisticated hybrid models have been proposed (e.g., Huser & Wadsworth, 2018; Wadsworth & Tawn, 2012; Wadsworth, Tawn, Davison, & Elton, 2017) spanning dependence classes, but these tend to be rather computationally challenging to estimate in practice.

The conditional extremes model of Heffernan and Tawn (2004) provides an alternative approach to characterising extremal spatial dependence admitting both AI and AD. The conditional extremes model also allows the incorporation of covariate effects (e.g., Jonathan, Ewans, & Randell, 2014). In the current work, we propose an extension of the conditional extremes method to a spatial setting, known as the spatial conditional extremes (SCE) model. SCE provides a framework to quantify the extreme marginal and dependence structure of *H*_*S*_ for locations in a neighbourhood, including the behaviour of extremal dependence of *H*_*S*_ at different locations as a function of the relative displacements of locations. Model estimation can be achieved using a relatively straightforward Markov chain Monte Carlo (MCMC) scheme and, unlike for MSP models, does not require composite likelihood techniques for parameter estimation; hence it does not incur parameter bias, as detailed in Tawn et al. (2018) and Wadsworth and Tawn (2018).

The layout of the article is as follows. In Section 2, we present motivating applications involving samples of *H*_*S*_ on spatial neighbourhoods in the northern North Sea (NNS) and the central North Sea (CNS). Section 3 outlines the SCE model. Parameter estimation is performed using Bayesian inference as described in Section 4; details of parameter constraints from Keef, Papastathopoulos, and Tawn (2013), and the Metropolis‐within‐Gibbs sampling scheme, are given in the Appendix. Results of the application of the SCE model to the north–south (N‐S) transect of the NNS sample are given in Section 5, with corresponding results for the east–west (E‐W) transect (for the NNS), and N‐S and E‐W transects for the CNS reported in Section 6. Section 7 provides discussion and conclusions.

## 2 MOTIVATING APPLICATION

**Figure 1:**
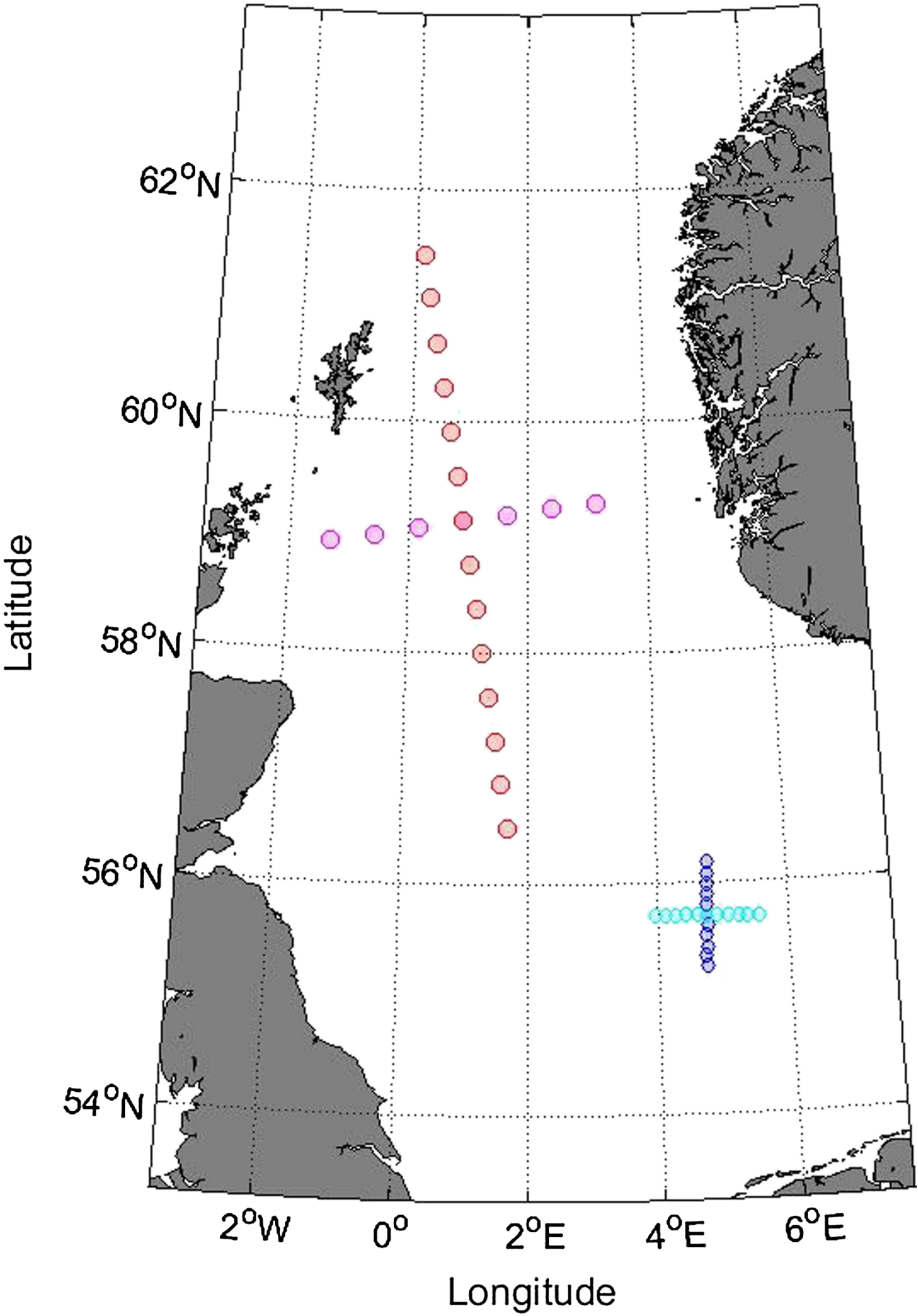
Northern North Sea and central North Sea locations considered

We consider hindcast data for storm peak significant wave height (henceforth *H*_*S*_ for brevity) from two neighbourhoods, one in the NNS and one in the CNS, as illustrated in Figure 1. In each neighbourhood, values for *H*_*S*_ are available on the N‐S and E‐W transects intersecting at a central location.

The NNS sample corresponds to winter storms (occurring in winter months October–March) from the NEXTRA hindcast (Oceanweather, 2002) for 20 locations on the two transects. Storm intervals for a total of 1,680 storms during the period Oct 1, 1964, to Mar 31, 1995, were isolated from up and down crossings of a sea state significant wave height threshold for the central location, using the procedure outlined in Ewans and Jonathan (2008). Storm peak significant wave height for each storm interval at each location provided a sample of 1,680 × 20 observations for further analysis. For each storm‐location combination, the direction (from which waves emanate, measured clockwise from North) at the time of the storm peak, referred to as the storm direction, was also retained. The spatial extremal characteristics of this sample have been examined previously in Ross et al. (2017); further discussion and illustrations of the data are available there.

The CNS sample corresponds to hindcast storm peak events (occurring at any time of year) for a period of 37 years from January 10, 1979, to December 30, 2015, for 21 locations on the two transects. The hindcast uses climate forecast system reanalysis (CFSR) wind fields (Saha et al., 2014) and a MIKE21 spectral wave simulator model (Sørensen, Kofoed‐Hansen, Rugbjerg, & Sørensen, 2005) to generate storm time series at each location. Storm periods were again identified as exceedances of a threshold nonstationary with respect to season and direction, using the procedure of Ewans and Jonathan (2008) for the central location. In this way, a total of 3,104 storm events were isolated per location for further analysis.

As will be explained further in Section 3, the SCE model is most conveniently considered for data with marginal standard Laplace distributions. For simplicity, we therefore choose to transform the NNS and CNS samples to standard Laplace scale prior to SCE analysis, as suggested by Keef, Tawn, and Lamb (2013), for example. This is achieved by estimating nonstationary marginal models (directional for NNS and directional‐seasonal for CNS), following the approach of Ross et al. (2017) and Ross, Randell, Ewans, Feld, and Jonathan (2017), independently per location. Transformed data then follow a standard Laplace distribution for each location.

Figure 1 illustrates that the interlocation spacing for the NNS hindcast is considerably larger than for the CNS hindcast. For this reason, it is important that we compare the variation of extremal spatial dependence between locations explicitly as a function of physical distance (here in kilometres). Scatterplots of Laplace‐scale storm peak *H*_*S*_ for pairs of locations separated by distances of 43.0, 171.8, and 300.7 km along the NNS N‐S transect (NNS:N‐S), coloured red in Figure 1, are shown by the black points in Figure 4 (see Section 5.1).

## 3 SPATIAL CONDITIONAL EXTREMES

### 3.1 Characterising extremal dependence

Key concepts in assessing extremal dependence are the notions of AD and AI. Typically, these are assessed through calculating two quantities, *χ* and $\bar{\chi }$, introduced by Coles et al. (1999). For bivariate data (*X*,*Y* ) with common margins, the quantity *χ* is calculated as

$$\chi =\underset{u\to u_{F}}{\lim}P(Y>u|X>u),$$

where *u*_*F*_ is the upper endpoint of the common marginal distribution *F* of the random variables. Then, $\bar{\chi }$ is defined by Coles et al. (1999) as $\bar{\chi }=2\eta -1$. Here, *η*, known as the coefficient of tail dependence, is defined by Ledford and Tawn (1996) from the asymptotic approximation, as *z*→*u*_*F*_,

$$P(X>z,Y>z)∼L\left(\frac{1}{P(X>z)}\right)\left[P{\left(X>z\right)}^{1/\eta }\right],$$

where *L*(*w*) is a slowly varying function so that *L*(*tw*)/*L*(*w*)→1 as *w*→*∞* for *t* > 0. Coles et al. (1999) provide details on how to calculate estimates for *χ* and $\bar{\chi }$. Then, *χ* > 0 defines the extent of AD present, whereas *χ* = 0 suggests that the variables exhibit AI. In the latter case, $\bar{\chi }$ measures the extent of AI present. Tawn et al. (2018) present a spatial equivalent for these measures. Crucially, the spatial characteristics under these two limiting extremal behaviour types are very different; under AD, two (or more) extreme events may occur at separate sites simultaneously, whereas under AI, this is not the case. Realistically, a spatial field is likely to exhibit a mixture of these behaviours: at short interlocation distance, AD may prevail; for sites at long distance apart, AI is more likely, leading to independence at very long distances. The SCE model accommodates both these possibilities.

### 3.2 The conditional extremes model of Heffernan and Tawn (2004)

In its simplest form, for a sample from a pair (*X*,*Y* ) of random variables with Laplace marginal distributions, for *x* larger than some suitable threshold *u*, the model proposed by Heffernan and Tawn (2004) is

(1) Y|{X=x}=a(x)+b(x)Z,

where *Z* is a residual process with typically unknown distribution function *G*. A particular form that may be utilised when working with Laplace margins is to set *a*(*x*) = *αx* and *β*(*x*) = *x* ^*β*^, for −1 ≤ *α* ≤ 1 and 0 ≤ *β* ≤ 1. This form of the conditional extremes model is used as the basis for the rest of this paper. We also assume that the unknown residual distribution *G* is Gaussian.

This model may be extended to a general multivariate case. Let *Z* be a multivariate Gaussian distribution with marginal distributions $N(\mu_{j},\sigma_{j}^{2})$ ( *j* = 0,…,*n*) for a set of spatial random variables (*X*_0_,…,*X*_*n*_) with standard Laplace margins. Then, we have a multivariate model given by

(2) $$(X_{1},\text{…},X_{n})|\{X_{0}=x\}∼\text{MVN}(\alpha x+\mu x^{\beta },B\Sigma B^{T}),$$

where *x* > *u*, ***α***=(*α*_1_,…,*α*_*n*_)^*T*^, ***β***=(*β*_1_,…,*β*_*n*_)^*T*^, ***μ***=(*μ*_1_,…,*μ*_*n*_)^*T*^, and $B=\text{diag}(x^{\beta_{1}},x^{\beta_{2}},\text{…},x^{\beta_{n}})$, and **Σ** is the variance–covariance matrix of the residuals *Z*. In expression (2), vector operations are carried out component‐wise.

We then have marginal models for *j* = 1,…,*n* given by

$$X_{j}|\{X_{0}=x\}∼N\left(\alpha_{j}x+\mu_{j}x^{\beta_{j}},\sigma_{j}^{2}x^{2\beta_{j}}\right).$$

Equation (2) corresponds to the multivariate extension of Equation (1), in which information about parameters $\theta ={\left\{\alpha_{i},\beta_{i},\mu_{i},\sigma_{i}\right\}}_{i=1}^{n}$ can be shared between random variables. The increased number of parameters, as compared to Equation (1), means that this model is more computationally challenging to estimate.

### 3.3 The SCE model

The SCE model is a spatial extension of the conditional extremes model, following the work of Tawn et al. (2018) and Wadsworth and Tawn (2018). First, suppose that *X*(·), the process of interest, is stationary and isotropic and has Laplace marginal distributions. In addition, suppose that we have sampling locations $s,s_{0}\in S$, where S is some spatial domain. Then, for *h* = |*s* − *s*_0_|, the distance or lag between two sites, we have

(3) $$X(s)|\{X(s_{0})>u\}=\alpha (h)X(s_{0})+X{\left(s_{0}\right)}^{\beta (h)}Z(s-s_{0}).$$

For a set of fixed spatial locations, Equations (2) and (3) are equivalent if we assume that *Z* is a residual Gaussian process with mean function *μ*(*h*) and covariance incorporating *σ*(*h*), as described in Equations (4) and (6). Of key importance is that different combinations of parameter values correspond to different types of spatial dependence. We have AD at all distances *h* when *α*(*h*) = 1 and *β*(*h*) = 0 for all *h* ≥ 0, whereas a mixture of limiting dependence classes is observed if (*α*(*h*),*β*(*h*)) = (1,0) for *h* ≤ *h*_AD_ but also *α*(*h*) < 1 for *h* > *h*_AD_, for some distance *h*_AD_. The process exhibits AD up to distance *h*_AD_ and AI thereafter. Hence, the proposed framework is able to estimate extremal dependence flexibly.

The model set out in expression (3) gives the behaviour of the process conditional on the process being extreme at *s*_0_. We need this model to hold for all $s_{0}\in S$ and for all of these conditional distributions to be self‐consistent with one another. Although the original multivariate conditional extremes models of Heffernan and Tawn (2004) do not impose additional assumptions about pairwise exchangeability, our choice of a stationary isotropic model imposes the required structure on the different conditional models to yield the required self‐consistency.

Although not key to developments in this paper, a natural question is whether the conditional models stem from a valid stochastic process. This is clarified by Wadsworth and Tawn (2018). They show that extreme events arising from a valid stochastic process can be generated over space, in such a way that events can be extreme at any spatial location. Therefore, although the SCE model is not explicitly specified as a stochastic process over space, it is specified implicitly for a process that has an extreme event somewhere in S. In this paper, we focus only on questions relating to the behaviour of the process given that there is an extreme event somewhere in S. Wadsworth and Tawn (2018) discuss an extension for which this condition is removed.

### 3.4 Constraints

For a given *h*, we constrain the possible values of pairs of parameters (*α*(*h*),*β*(*h*)), as suggested by Keef et al. (2013) and outlined in the Appendix. The motivation for this constraint is to impose an ordering of conditional distributions associated with AI (*α*(*h*) < 1) and asymptotic positive dependence (*α*(*h*) = 1,*β*(*h*) = 0). In practice, this means that certain combinations of (*α*(*h*),*β*(*h*)) are inadmissible.

We also impose gradient‐based constraints on (*α*(*h*),*β*(*h*)) following Lugrin, Davison, and Tawn (2018), in order to improve the identifiability of the parameter combinations. The motivation for these constraints is ensuring that the derivative, with respect to *x*, of $E(X(h)|X(0)=x)=\alpha (h)x+\mu (h)x^{\beta (h)}$ is positive, for *x* ≥ *u*, with *u* some suitable threshold; we then have the constraints *α*(*h*) + *μ*(*h*)*β*(*h*)*x* ^*β*(*h*) − 1^ ≥ 0 and *α*(*h*) ≥ 0 for all *h*.

## 4 INFERENCE

We consider two variants of the SCE model, differing by the manner in which “linear slope” parameters {*α*_*k*_} are estimated. In the more general form, outlined in Section 4.1, these parameters are estimated freely given the sample data, likelihood function, and constraints from Section 3.4. In the restricted parametric form, outlined in Section 4.2, the decay of *α* with distance *h* follows a prescribed physically plausible exponential form described by only two parameters. We first consider the more general “free” model.

### 4.1 Likelihood for the “free” model

**Figure 2:**
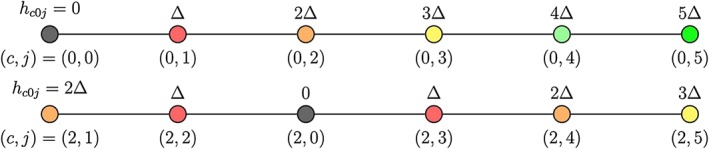
Illustration of notation used to describe the disposition of points on the line, enabling pooling of data from pairs of locations by distance. The (*c*,*j*) notation is shown below the line, and distance *h*_*c*0*j*_ is given above each point. Points with the same value of *h*_*c*0*j*_ are shown in the same colour; Δ is the interlocation spacing

Consider *p* + 1 equally spaced points on a transect. Suppose we condition on the value of *H*_*S*_ at a point on the line, marked in black in the two examples of Figure 2. Our goal is to fit a joint distribution for the values of *H*_*S*_ at all remaining points, conditioned on an extreme value observed at the conditioning point.

As the set of remaining random variables depends on the conditioning point chosen, we require two indices to define locations: an index *c* ∈ {0,1,2,…,*p*} to indicate the “conditioning” point and an index *j* ∈ {1,2,…,*p*} for the remaining points on the line, which we henceforth call “remote” points. The conditioning point will therefore always have an index of the form (*c*,0), as illustrated in Figure 2, where *c* = 0 in the upper image and *c* = 2 in the lower image.

We indicate the location of the conditioning point as *s*_*c*0_ and the location of remote points using {*s*_*cj*_}. The distances of remote points to the conditioning point are then denoted by {*h*_*c*0*j*_}, with *h*_*c*0*j*_ = |*s*_*cj*_ − *s*_*c*0_|. Similarly, the distances between remote points (*c*,*j*) and (*c*,*j* *′*) are denoted $\left\{h_{cjj^{\prime }}\right\}$ with $h_{cjj^{\prime }}=|s_{cj^{\prime }}-s_{cj}|$; example values of (*c*,*j*) and *h*_*c*0*j*_ are indicated in Figure 2. In the case of the lower image in Figure 2, note that there are locations that sit a common distance from the conditioning point (with the same value of *h*_*c*0*j*_, shown as discs of the same colour).

We assume that conditional dependence is isotropic on a transect so that the parameters of the SCE model are at most a function of interlocation distances only. Specifically, the parameters *α*, *β*, *μ*, and *σ* are functions of distance from conditioning location, and the residual dependence between remote locations will in addition be a function of distances between remote locations. We seek a model for the joint dependence structure for any number of locations conditional on an extreme value at the conditioning location. For definiteness, consider first the case of two remote locations (*c*,*j*) and (*c*,*j* *′*) (with *j* *′* ≠ *j*) and conditioning location (*c*,0), and corresponding random variables $\left(X_{cj},X_{cj^{\prime }},X_{c0}\right)$. We can then write the SCE model as

(4) $$(X_{cj},X_{cj^{\prime }})|\{X_{c0}=x_{c0}\}∼{\text{MVN}}_{2}\left(M_{cjj^{\prime }},C_{cjj^{\prime }}\right),\;x_{c0}>q_{\tau },$$

where *q*_*τ*_ is the quantile of a standard Laplace distribution with nonexceedance probability *τ*,

$$M_{cjj^{\prime }}=[\alpha (h_{c0j}),\alpha (h_{c0j^{\prime }})]x_{c0}+[\mu (h_{c0j}),\mu (h_{c0j^{\prime }})]x_{c0}^{\left[\beta (h_{c0j}),\beta (h_{c0j^{\prime }})\right]}$$

and

(5) $$\begin{array}{ll} C_{cjj^{\prime }} & =\left[\begin{array}{cc} x_{c0}^{\beta (h_{c0j})} & 0\\ 0 & x_{c0}^{\beta (h_{c0j^{\prime }})} \end{array}\right]\left[\begin{array}{cc} \sigma (h_{c0j}) & 0\\ 0 & \sigma (h_{c0j^{\prime }}) \end{array}\right]\left[\begin{array}{cc} 1 & \rho^{h_{cjj^{\prime }}}\\ \rho^{h_{cjj^{\prime }}} & 1 \end{array}\right]\\ & \;\times {\left[\begin{array}{cc} \sigma (h_{c0j}) & 0\\ 0 & \sigma (h_{c0j^{\prime }}) \end{array}\right]}^{T}{\left[\begin{array}{cc} x_{c0}^{\beta (h_{c0j})} & 0\\ 0 & x_{c0}^{\beta (h_{c0j^{\prime }})} \end{array}\right]}^{T}, \end{array}$$

and *ρ* is the between‐neighbour residual correlation parameter. We can extend the model to three or more remote locations, or reduce it for one remote location in the obvious way. Hence, we can construct a sample Gaussian likelihood *L* under the model for all observations, with a conditioning variate exceeding *q*_*τ*_, of all possible combinations of two or more locations on the line. We note that, in Equation (5), any correlation function *K*(·) could be used in the third matrix; for this work, we specifically use an exponential correlation function so that $K(h_{cjj^{\prime }})=\rho^{h_{cjj^{\prime }}}$.

The likelihood *L* is a function of {*α*(*h*_*c*0*j*_), *β*(*h*_*c*0*j*_), *μ*(*h*_*c*0*j*_), *σ*(*h*_*c*0*j*_)}, and *ρ* (for different distances $\left\{h_{cjj^{\prime }}\right\}$ between remote locations). Because the locations are equally spaced, the values of *α*, *β*, *μ*, and *σ* can only be estimated for given distances *h* = *k*Δ, for lag index *k* = 1,2,…,*p*, where Δ is the location spacing for the application (expressed in kilometres). For ease of discussion below, we can therefore write L≜L(θ) for the full parameter set as

(6) $$\theta =\left\{{\left\{\alpha_{k},\beta_{k},\mu_{k},\sigma_{k}\right\}}_{k=1}^{p},\rho \right\},$$

where parameters are indexed by lag *k* not distance *h* so that *α*_*k*_ = *α*(*k*Δ), et cetera.

In practice, we also pool all available observations corresponding to unique combinations of distances (i.e., from different choices of conditioning location (*c*,0)) in the SCE likelihood; we thereby exploit the sample well, in a computationally favourable manner. Hence, we no longer have the true likelihood under our model but, instead, a pseudolikelihood because the same observation (of each location on a transect) may enter more than one conditioning likelihood contribution (corresponding to conditioning on extreme values at a particular location). Using a pseudolikelihood as if it is a likelihood is widely known to give point estimates that are asymptotically consistent, but those measures of uncertainty are underestimated. In our Bayesian inference, we expect to underestimate posterior credibility intervals using these pooled data.

Various approaches are available to adjust estimated uncertainty, either by inflating variances or modifying the pseudolikelihood. In Bayesian inference, the methods of Ribatet, Cooley, and Davison (2012) provide an appropriate approach to valid inference for any selected model. In this paper, however, we use the raw pseudolikelihood for presentation of results, which we justify as follows. The paper focuses on model selection between the free model introduced in this section and a nested parametric model, introduced in Section 4.3, with the actual uncertainties of the parameters being of secondary importance relative to the selection of the better model. When using the pseudolikelihood in place of the full likelihood, inference for the free model will give parameter estimates with credible intervals that are too narrow. Thus, if our subsequent parametric model estimates fall inside these intervals, it suggests that the parametric model provides a better fit than the free model. We emphasise that credible intervals referred to in this work correspond to pseudolikelihood credible intervals.

### 4.2 MCMC for the free model

We use Bayesian inference to estimate the joint posterior distribution of parameters *θ* from Equation (6). In our experience, Bayesian inference with reasonable prior specification and MCMC scheme provides a more reliable approach to parameter estimation than maximum likelihood techniques. An outline of the procedure, discussion of the priors used, and an algorithm are given in the Appendix. Briefly, we proceed as follows.

First, we use random search to find a reasonable starting value for *θ*. Then, to improve on the starting solution, we use a Metropolis‐within‐Gibbs algorithm iteratively to sample each of the elements of *θ* in turn. Then, we use a *grouped adaptive* random walk Metropolis‐within‐Gibbs algorithm iteratively to convergence, judged to have occurred when trace plots for parameters and their dependence stabilise. Within the grouped adaptive algorithm, we jointly update the parameters (*α*_*k*_,*β*_*k*_,*μ*_*k*_,*σ*_*k*_) for each *k*, following the adaptive approach of Roberts and Rosenthal (2009) to make correlated proposals. We also adjust proposal standard deviation such that the acceptance rate is optimised for all parameters.

### 4.3 Inference for the “parametric‐***α***” model

Though the constraints of Section 3.4 go some way in improving identifiability of suitable parameter combinations, it is still difficult to obtain plausible results in some cases for the free SCE model. Therefore, we shall consider a parametric form for *α* based on physical considerations, whereby *α*(*h*) in general should not only decrease with increasing *h* but also reduce the dimension of the parameter space, helping parameter identifiability. Specifically, we explore the performance of an SCE model where *α* is parameterised as a function of distance, writing

(7) $$\alpha_{k}=\exp\left\{-{\left(\frac{k}{\kappa_{1}}\right)}^{\kappa_{2}}\right\},\;k=1,2,\text{…},p$$

with parameters *κ*_1_,*κ*_2_ > 0. The resulting likelihood is $L≜L(\theta^{*})$ with adjusted parameter set

(8) $$\theta^{*}=\left\{\kappa_{1},\kappa_{2},{\left\{\beta_{k},\mu_{k},\sigma_{k}\right\}}_{k=1}^{p},\rho \right\}.$$

The MCMC procedure for the parametric‐*α* model is similar to that for the free model, except that *κ*_1_, *κ*_2_ are separated from the grouped parameters (*β*_*k*_,*μ*_*k*_,*σ*_*k*_) for each *k*.

### 4.4 Comparison of free and parametric‐*α* models

To compare results from free and parametric‐*α* models, we use the deviance information criterion (DIC), as proposed by Spiegelhalter, Nicky, and Carlin (2002), a Bayesian analogue of the Akaike Information Criterion (Akaike, 1974). Defining D(θ)=−2logL(θ), where *L* is our pseudolikelihood, we measure model complexity using

$$p_{D}=\bar{D(\theta )}-D(\bar{\theta }),$$

where $\bar{D(\theta )}$ is the average of the deviances (calculated after burn‐in) and quantifies lack of fit. Furthermore, $\bar{\theta }$ is the average of posterior estimates of *θ*; note that this is an estimate for the posterior mean. Explicitly, from the final *m* iterations of the MCMC chain, we calculate

$$\bar{\theta }=\frac{1}{m}\overset{m}{\underset{i=1}{\sum }}\theta^{\left(i\right)}\;\text{and}\;\bar{D(\theta )}=\frac{1}{m}\overset{m}{\underset{i=1}{\sum }}D(\theta^{\left(i\right)}),$$

where component‐wise averages are taken in the first equation. The DIC is then calculated as

$$\text{DIC}=p_{D}+\bar{D(\theta )}=2\bar{D(\theta )}-D(\bar{\theta }),$$

with lower values preferred.

## 5 APPLICATION TO THE NNS N‐S TRANSECT (NNS:N‐S)

We now apply the free model and parametric‐*α* model to data for the NNS:N‐S transect. We start by considering the free model in some detail (in Section 5.1), demonstrating that the fitted model explains the data well. Next, in Section 5.2, we consider the corresponding parametric‐*α* model and show that this also fits well, as well as using the DIC, as defined in Section 4.4, to show that the fit of free and parametric‐*α* models is similar. The analysis is extended to other transects and locations in Section 6. Throughout this section, we adopt a conditioning threshold with nonexceedance probability *τ* = 0.9 for the SCE model, after testing the stability of inferences to other choices of threshold. Threshold choice of course involves a bias‐variance trade‐off: increasing sample size for tail modelling versus inclusion of points from outside the tail region. We note that parameter estimates were relatively stable for choices of extreme value threshold above *τ* = 0.8 and below either *τ* = 0.9 (for NNS data) or *τ* = 0.95 (for CNS data).

### 5.1 Free model

**Figure 3:**
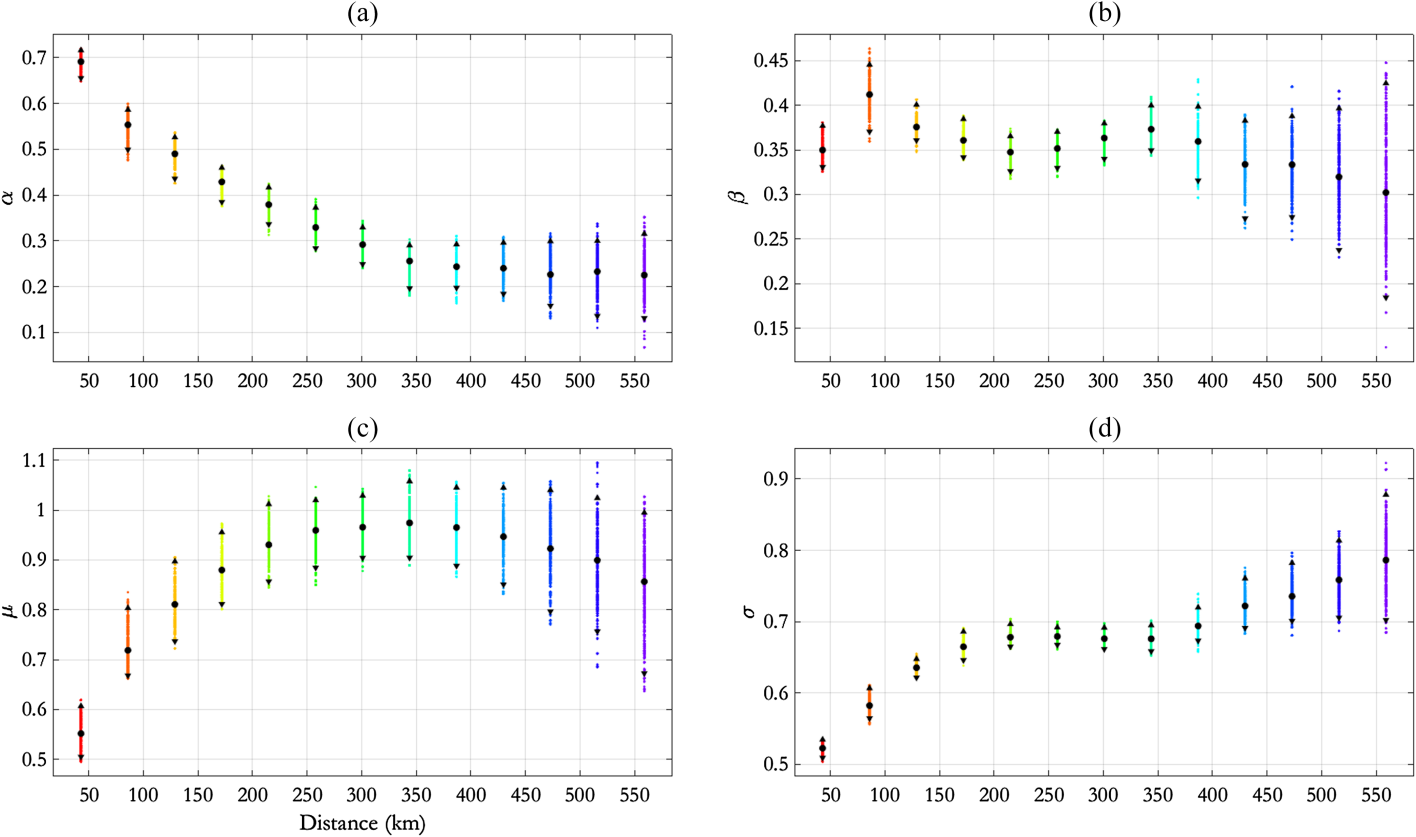
NNS:N‐S transect, free model: parameter estimates for (a) *α*, (b) *β*, (c) *μ*, and (d) *σ* with distance *h*, summarised using posterior means (disc) and 95% pseudolikelihood credible intervals (with end points shown as solid triangles). NNS = northern North Sea; N‐S = north–south

The inference scheme introduced in Section 4 is used to estimate parameters *θ* (see Equation (6)) for the NNS:N‐S transect. Posterior mean and pseudolikelihood credible intervals for estimates of each of *α*(*h*), *β*(*h*), *μ*(*h*), and *σ*(*h*) from the final 1,000 iterations (out of a total of 20,000 iterations) of the MCMC algorithm described in Section 4.2 are shown in Figure 3. Trace plots showing convergence of MCMC chains are given in the Supplementary Material accompanying this article. We note that the parameter *ρ* has a posterior mode of approximately 0.73 and a 95% pseudolikelihood credible interval with width of approximately 0.09.

**Figure 4:**
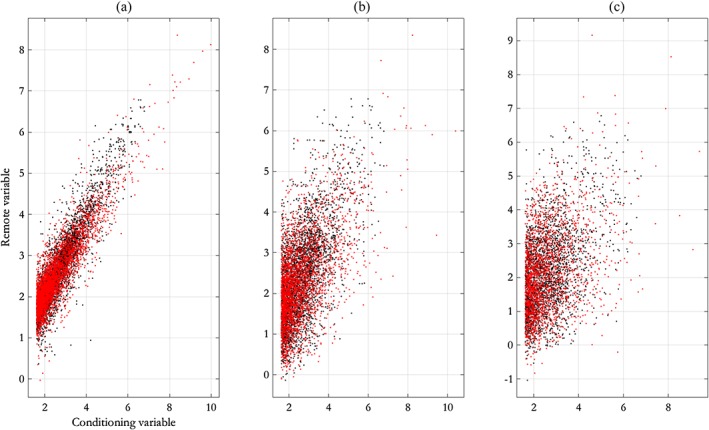
Scatterplots illustrating dependence between values of Laplace‐scale storm peak *H*_*S*_ at different relative distances for NNS:N‐S transects, from (a) original sample and (b) simulation under the fitted free model. Black points are the original data on Laplace scale; red points are data simulated under the fitted model. NNS = northern North Sea; N‐S = north–south

We see from Figure 3 that *α* decays exponentially with *h*; this motivates the adoption of the parametric‐*α* model in Section 5.2. In particular, we see that *α*(*h*) ≠ 1 for any *h*, so this suggests that AI is present for all distances *h*. We see that *μ*(*h*) mirrors the behaviour of *α*(*h*) to some extent in that, for *h* < 200 km, *μ* increases fairly quickly before stabilising and possibly decreasing again; this illustrates the anticipated dependence between estimates for *α* and *μ* in the conditional extremes model. The parameter *β* is relatively constant with *h*, taking values between 0.3 and 0.4, whereas *σ* increases in general with *h*. The behaviour of *α*(*h*) and *σ*(*h*) appears reasonable given physical intuition and evidence from the data (the black points) in Figure 4: Extremal dependence reduces as distance between conditioning and remote sites increases, yet the overall variability at each location is constant given that *H*_*S*_ at each location has been transformed to the standard Laplace scale.

**Figure 5:**
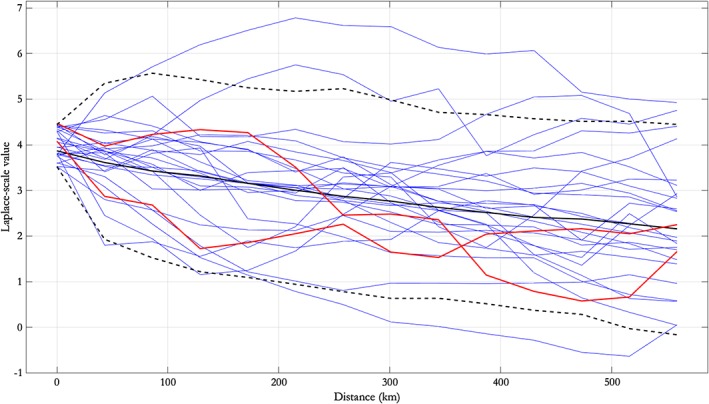
Observed spatial processes from the NNS:N‐S transect with Laplace‐scale values at the left‐hand location in the interval [3.5,4.5], together with posterior predictive estimates from simulation under the fitted free model, represented using the median (black) and upper and lower limits of a 95% pseudolikelihood credible interval. Red lines are simulated spatial processes from the fitted model

Figures 4 and 5 display diagnostics for the fitted model. Figure 4 shows the original data on the Laplace scale (in black) at three different separations *h* of remote and conditioning points. Data simulated under the fitted model are overlaid in red; there is good general agreement. Figure 5 shows observed sequences of *H*_*S*_ values along transects with conditioning value (of *H*_*S*_ at either end point of the transect) between 3.5 and 4.5 on Laplace scale in blue, as well as two simulated spatial processes from the fitted model, shown in red. The figure also shows the corresponding 95% pseudolikelihood credible interval under the fitted SCE model with conditioning values between 3.5 and 4.5; again, there is general agreement between observation and simulation under the fitted model; in particular, the simulated processes appear to have similar smoothness to the observed processes.

### 5.2 “Parametric‐***α***” fit

Figure 3 suggests an exponential decay of parameter *α* with distance *h* in the free model. Here, we examine the performance of the SCE model with the functional form for *α*(*h*) given in Eq. (7) and with parameters *θ* ^∗^ to estimate (as defined in (8)).

**Figure 6:**
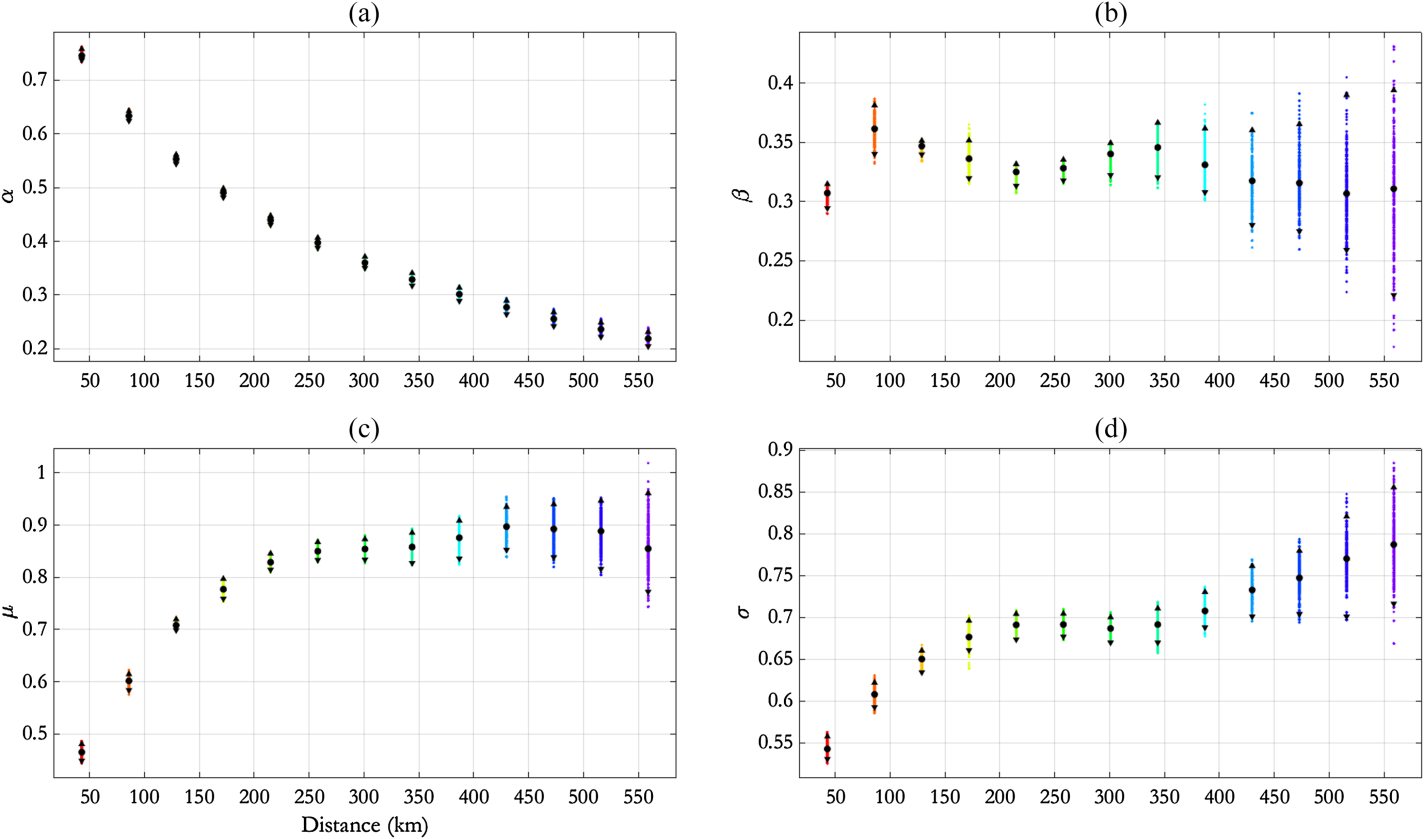
NNS:N‐S transect, parametric‐*α* model: parameter estimates for (a) *α*, (b) *β*, (c) *μ*, and (d) *σ* with distance *h*, summarised using posterior means (disc) and 95% pseudolikelihood credible intervals (with end points shown as solid triangles). NNS = northern North Sea; N‐S = north–south

Comparing Figures 3 and 6 shows that pseudolikelihood credible intervals for *α*(*h*) are considerably narrower in the parametric‐*α* model. This is not surprising because the parametric‐*α* model has a smaller number of parameters. Moreover, the parametric decay of *α* in the parametric‐*α* model restricts its possible values for any *h*. Furthermore, they show that posterior mean estimates for *α*(*h*), *β*(*h*), *μ*(*h*), and *σ*(*h*) are similar under the two models.

The informal discussion above suggests that the quality of fit of the free and parametric‐*α* models is similar. To compare these models more formally, we use the DIC introduced in Section 4.4. Values for parameter estimates and likelihood from the last *m*=1,000 MCMC iterations are used to estimate the DIC for the two models; the DIC for the free model was calculated to be 27,514.22, and for the parametric‐*α* model, 27,501.68. Because the DIC for the parametric‐*α* model is smaller than that for the free model, we infer in this case that the parametric‐*α* model is to be preferred and that the difference between free and parametric‐*α* fits is small. However, the parametric‐*α* model has the additional advantage that the computational time is decreased due to the smaller number of parameters to estimate in this version of the SCE model.

## 6 APPLICATION TO OTHER NORTH SEA TRANSECTS

The wave environment in the NNS and CNS is known not to be isotropic (e.g., Feld, Randell, Wu, Ewans, & Jonathan, 2015); we might therefore suspect that the extremal spatial dependence in these neighbourhoods might also be sensitive to transect orientation. Inspection of Figure 1 shows that fetches in the NNS are in general longer than in the CNS; furthermore, water depths in the NNS are greater than those in the CNS. It is not unreasonable therefore to anticipate that extremal spatial dependence may be different in different regions of the North Sea. Moreover, for the data considered here, the CNS data are available on a finer grid than for the NNS data, so we may be able to pick out finer‐scale features of the dependence structure. Furthermore, the lengths of transects and their spatial resolutions vary, offering the possibility of detecting finer‐scale effects (in the CNS) and longer‐range effects (for transects with largest distances *h*). This motivates estimating SCE models for the NNS:E‐W transect, and the CNS:N‐S and CNS:E‐W transects.

Below, we start by comparing DIC values for the free and parametric‐*α* models. Because it was found that the performance and characteristics of the models were similar for all transects, subsequent discussion of parameter behaviour with *h* is restricted to the parametric‐*α* model. As in Section 5, all MCMC chains are of length 20,000, and we utilise the final 1,000 iterations for inference.

### 6.1 Comparison of model fits for all transects

We compare DIC values for free and parametric‐*α* model parameterisations to assess in particular whether the parametric‐*α* model is a reasonable general representation for all transects, relative to the free model. Table 1 gives values for the DIC for each of the transects considered in this work.

**Table 1:** Table of deviance information criterion (DIC) values for the free fit model and the parametric‐*α* model for all of the transect analyses

Model	DIC	
	**Free**	**Parametric**‐*****α*****
NNS:N‐S	27,514.22	27,501.68
NNS:E‐W	7,360.93	7,356.75
CNS:N‐S	23,471.67	23,476.13
CNS:E‐W	23,809.94	23,827.10

*Note*. NNS = northern North Sea; N‐S = north–south; E‐W = east–west; CNS = central North Sea.

From the table, we see that the DIC is lower for the parametric‐*α* model for NNS transects; for the CNS transects, the free model produces lower values for the DIC. However, comparing the differences between DIC values per transect with the variability of the corresponding negative log‐likelihoods from the MCMC, we see that differences in the DIC are small in each case. We conclude that there is little material difference between free and parametric‐*α* fits for any of the transects.

### 6.2 NNS E‐W transect

**Figure 7:**
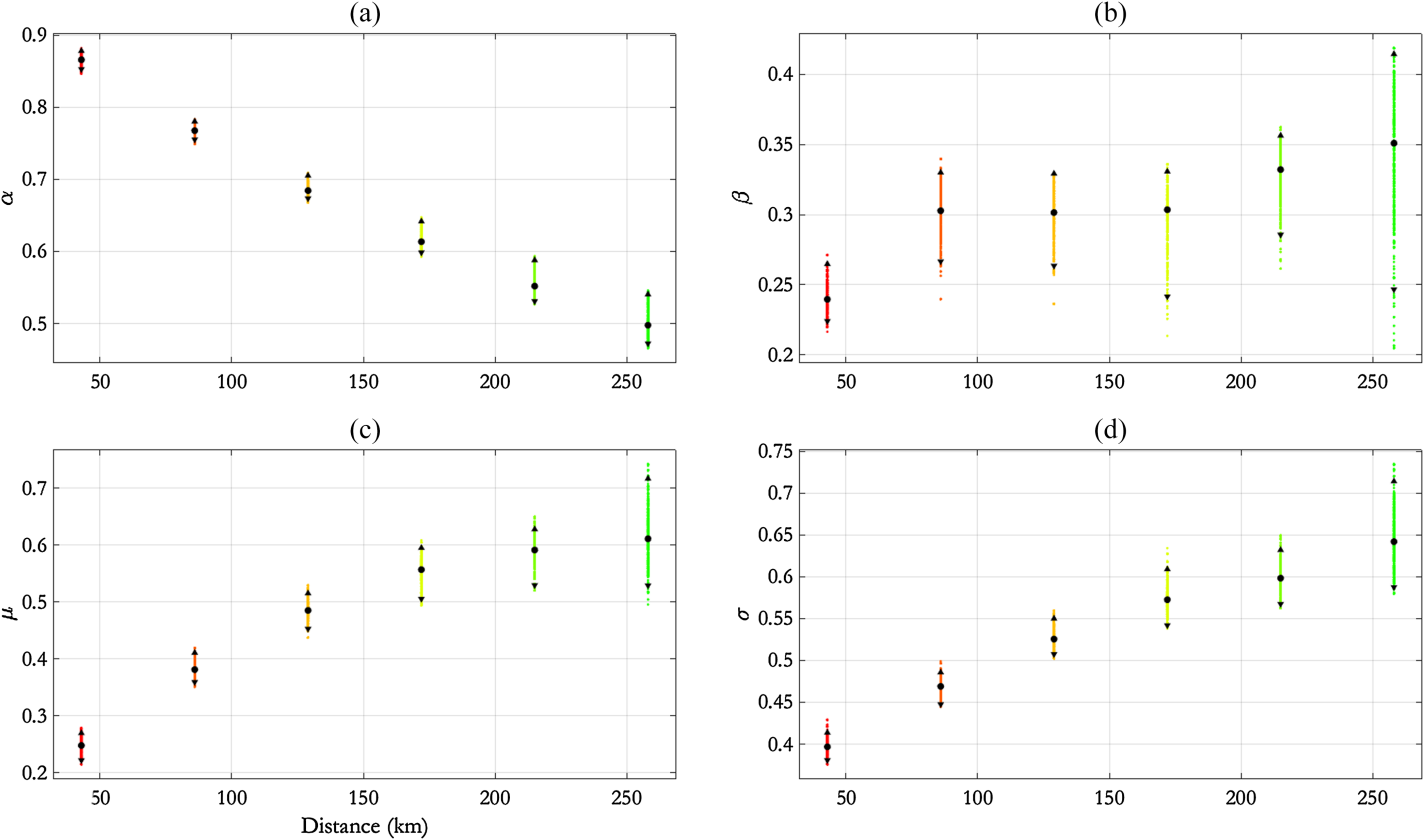
NNS:E‐W transect, parametric *α*(*h*) model: estimates for (a) *α*(*h*), (b) *β*(*h*), (c) *μ*(*h*), and (d) *σ*(*h*) with distance *h*, summarised using posterior means (disc) and 95% pseudolikelihood credible intervals (with end points shown as solid triangles). NNS = northern North Sea; E‐W = east–west

We first apply the parametric‐*α* model to NNS:E‐W, coloured magenta in Figure 1, using a nonexceedance probability of *τ* = 0.9 when applying the SCE model, as in Section 5. Posterior estimates for model parameters are shown in Figure 7. This transect has fewer sites available for analysis than NNS:N‐S in Section 5, and hence, fewer data may be pooled together for estimation. Therefore, we would naturally expect model parameter uncertainties to be larger. From the figure, it is clear that the pseudolikelihood credible intervals are wider than for NNS:N‐S, at similar *h*.

The behaviour of parameter estimates for *μ* and *σ* with *h* are similar to those observed for NNS:N‐S. However, in NNS:E‐W, *β* increases with distance. The figure also illustrates that estimates for *α*(*h*) on NNS:E‐W are larger; in particular, *α*(*h*≈50 km)≈0.9, suggesting that dependence is much higher at short range for NNS:E‐W than for NNS:N‐S, for which *α*(*h*≈50 km)≈0.6. Furthermore, the rate of decay of *α* with *h* is smaller for NNS:E‐W than for NNS:N‐S. These findings are plausible given physical intuition: The largest events in the NNS are Atlantic storms travelling approximately E‐W. It is reasonable then to expect that spatial dependence along E‐W transects may be higher than for transects with other orientations.

### 6.3 CNS transects

**Figure 8:**
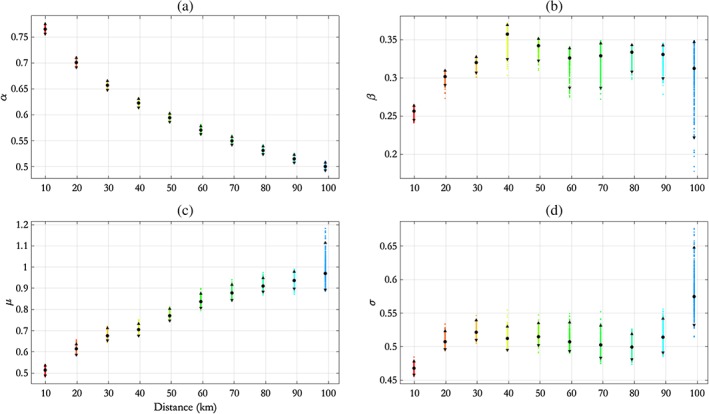
CNS:N‐S transect, parametric‐*α* model: parameter estimates for (a) *α*, (b) *β*, (c) *μ*, and (d) *σ* with distance *h*, summarised using posterior means (disc) and 95% pseudolikelihood credible intervals (with end points shown as solid triangles). CNS = central North Sea; N‐S = north–south

For the CNS N‐S transects (CNS:N‐S, coloured dark blue in Figure 1, and CNS:E‐W, coloured cyan), the separation Δ of locations is smaller than for NNS transects. Furthermore, as more data are available at each site for this ocean basin, we set *τ* = 0.95 for the SCE model. Parameter estimates from the parametric‐*α* model are shown in Figure 8 for CNS:N‐S.

Compared to NNS transects, *α* decreases quickly with *h*. At *h*≈100 km, the value of *α* is approximately 0.5, close to that estimated for the NNS:N‐S transect at *h*≈150 km, but at *h*≈250 km for NNS:E‐W. The behaviour of *μ* and *σ* with *h* is similar to earlier cases, and *β* is approximately constant at approximately 0.3, and *σ* at 0.52. Pseudolikelihood credible intervals for estimates increase with *h*.

**Figure 9:**
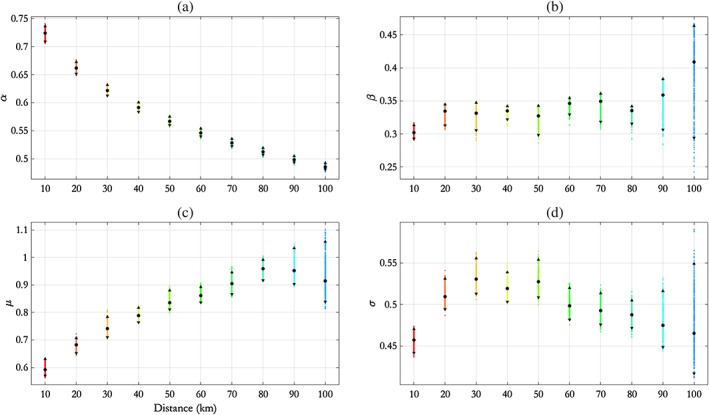
CNS:E‐W transect, parametric‐*α* model: parameter estimates for (a) *α*, (b) *β*, (c) *μ*, and (d) *σ* with distance *h*, summarised using posterior means (disc) and 95% pseudolikelihood credible intervals (with end points shown as solid triangles). CNS = central North Sea; E‐W = east–west

For the CNS:E‐W transect, posterior estimates for the SCE parameters are shown in Figure 9; this transect is slightly longer than the CNS:N‐S transect.

The parameter *μ* increases with *h*, and *β* is approximately constant at approximately 0.33. There is some evidence that *σ*(*h*) decreases for *h* > 50 km. The general behaviour of *α* with *h* is similar to that for the CNS:N‐S transect, with a somewhat slower decay.

## 7 DISCUSSION AND CONCLUSIONS

In this work, we use an SCE model to investigate the extremal dependence of significant wave height *H*_*S*_ along straight line transects of different lengths with different spatial orientations and resolutions in NNS and CNS. The analyses described in Sections 5 and 6 suggest that the general nature of extremal dependence is similar for all transects. It appears that the linear dependence parameter *α* in the SCE model decays with separation *h* of locations and that this decay is approximately exponential (recalling that *H*_*S*_ is expressed on standard Laplace scale). The parameter *μ* increases with *h*, potentially to a finite asymptote, whereas the parameter *β* appears to remain approximately constant as a function of *h*. There is some evidence that *σ* increases initially with *h*, but no consistent subsequent behaviour is observed.

**Figure 10:**
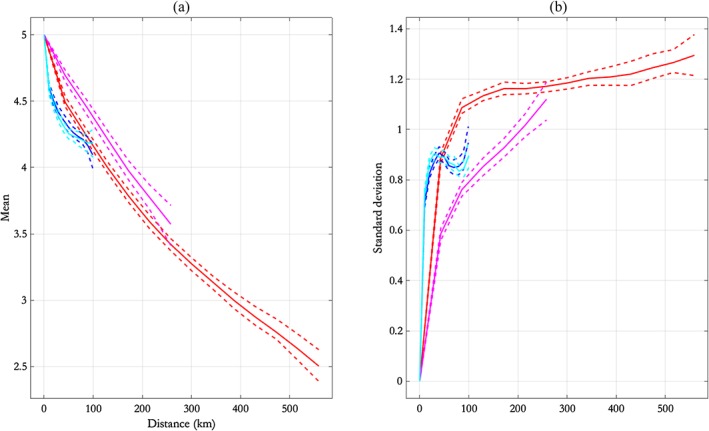
Pseudolikelihood credible intervals for (a) the conditional mean and (b) the conditional standard deviation of the fitted dependence model as a function of distance in kilometres, for conditioning Laplace‐scale value of 5, and the different transects, NNS:N‐W (red), NNS:E‐W (magenta), CNS:N‐S (blue), and CNS:E‐W (cyan). NNS = northern North Sea; N‐S = north–south; E‐W = east–west; CNS = central North Sea

Features of the extremal dependence vary by region and transect orientation. For instance, we note that the estimate of *ρ*, the residual dependence parameter, for the NNS:N‐S transect (with a posterior mode of approximately 0.73 and a 95% pseudolikelihood credible interval width of approximately 0.06) is different from its value for the other three transects (for which *ρ* is estimated to have a mode of approximately 0.5 in each case, and 95% pseudolikelihood credible intervals of width of approximately 0.06). Figure 10a illustrates the behaviour of the conditional mean *α*(*h*)*x* + *μ*(*h*)*x* ^*β*(*h*)^ from the SCE model for a (Laplace‐scale) conditioning value *x* = 5, approximately corresponding to the 0.997 quantile. Figure 10b shows the corresponding evolution of the conditional standard deviation *σ*(*h*)*x* ^*β*(*h*)^.

From Figure 10, it is clear that extremal dependence of *H*_*S*_ in the NNS is more persistent than in the CNS, and that extremal dependence on the NNS:E‐W transect is more persistent than on the NNS:N‐S transect (see also Section 6.2). That is, longer‐range extremal dependence is observed for the E‐W transect in the NNS; the same conclusion was drawn by Ross et al. (2017) in their analysis of related data for the same region, using one‐ and two‐dimensional MSP models. It will be interesting to extend the current SCE model to two‐dimensional neighbourhoods of locations, particularly to investigate whether directional differences, related to differences due to transect orientation reported here, are observed.

From an intuitive perspective, we expect the value of SCE parameter *α* to decay to zero for large *h* because, for large *h*, the value at the conditioning location should not affect the value at the remote location. For the same reason, we expect *β*(*h*) and *μ*(*h*) to decay to zero, and *σ*(*h*) to asymptote to a finite value; see Wadsworth and Tawn (2018) for discussion of the modelling of spatial independence at long range. We plan to examine this by exploring the characteristics of storm peak *H*_*S*_ on long transects extending over at least 1,000 km.

Inspection of Equation (2) or (5) readily shows that identification of SCE model parameters is problematic in general, although considerations such as those of Keef et al. (2013) help restrict the admissible set of parameter values. Imposing an exponential form on the decay of *α*(*h*) with *h* was found at least not to be detrimental in the current work. Inspection of the resulting behaviour of parameter estimates in the figures above suggests that further parameterisation of *μ*(*h*) in particular may be useful.

Understanding the extremal spatial dependence of ocean storms is important for the reliable characterisation of extreme storms and their impact on marine and coastal facilities and habitats. From a statistical perspective, the ocean environment provides a useful test bed for models for spatial extremal dependence over a range of distances. From an offshore engineering perspective, the findings of studies such as the present work can lead to more informed procedures to accommodate the effects of spatial dependence in engineering design guidelines. The SCE model would seem to offer a relatively straightforward method to help achieve this.

## Supporting information

ENV_2562‐Supp‐0001‐Online supporting information.pdf

## APPENDIX 1

### 1.1

This appendix summarises the constraints on the conditional extremes model introduced by Keef et al. (2013) and the MCMC procedure used for parameter estimation.

### THE CONSTRAINTS OF KEEF ET AL. (2013)

We also constrain the possible parameter values of *α*(*h*) and *β*(*h*), for *h* > 0, as suggested by Keef et al. (2013). The constraint of interest, for *α*(*h*), *β*(*h*), and some given *h*, in this work is Case 1 of Theorem 1, as given by Keef et al. (2013), namely, that we require either

$$\alpha (h)\leq \min\{1,1-\beta (h)z_{h}(q)v^{\beta (h)-1},1-v^{\beta (h)-1}z_{h}(q)+v^{-1}z_{h}^{+}(q)\}$$

or

$$\begin{array}{ll} & 1-\beta (h)z_{h}(q)v^{\beta (h)-1}<\alpha (h)\leq 1,\;\text{and}\\ & (1-\beta {\left(h\right)}^{-1}){\left\{\beta (h)z_{h}(q)\right\}}^{1/(1-\beta (h))}{\left(1-\alpha (h)\right)}^{-\beta (h)/(1-\beta (h))}+z_{h}^{+}(q)>0. \end{array}$$

Here, *z*_*h*_(*q*) is the *q*th quantile of the distribution of standardised residuals from the conditional extremes model at distance *h* with nonexceedance probability *q*. Similarly, $z_{h}^{+}(q)$ is the *q*th quantile of the distribution of standardised residuals from the conditional extremes model assuming asymptotic positive dependence (i.e., forcing *α*(*h*) = 1, *β*(*h*) = 0) at distance *h* with nonexceedance probability *q*. In practice, as suggested by Keef et al. (2013), it is sufficient to satisfy the constraints above for *q* = 1 and *ν* equal to the maximum observed value of the conditioning variate.

### MCMC PROCEDURE

The MCMC method implemented in Section 4.2 is adapted from the method of Roberts and Rosenthal (2009). Suppose the parameters of interest are $\theta ={\left\{\alpha_{k},\beta_{k},\mu_{k},\sigma_{k}\right\}}_{k=1}^{p}\cup \{\rho \}$, where *p* is the number of sampling locations. The total number of parameters is therefore *n*_*P*_ = 4*p* + 1. We impose uniform prior distributions for each of these parameters; explicitly, *π*(*α*_*k*_)∼Unif(0,1), *π*(*β*_*k*_)∼Unif(0,1), *π*(*μ*_*k*_)∼Unif( − 2,2) and *π*(*σ*_*k*_)∼Unif(0,3) for all *k* = 1,…,*p*, and *π*(*ρ*)∼Unif(0,1). A total of *n* updates of ***θ*** will be performed.

First, we obtain a random starting solution ***θ*** ^(0)^ by sampling the elements of ***θ*** from their prior distributions, verifying that the starting solution has a valid likelihood (defined in Section 4.1).

Writing $\theta_{k}^{\left(i\right)}$ as the value of the *k*th parameter of ***θ*** at the *i*th iteration; we then use an adaptive random walk Metropolis‐within‐Gibbs scheme for *n*_*S*_ iterations. That is, for *i* = 2,…,*n*_*S*_, where *n*_*S*_ < *n*, we update each $\theta_{k}^{\left(i\right)}$ in turn. If *i* ≤ 2*n*_*P*_, we propose a candidate value $\theta_{k}^{(i)c}$ from distribution

$$Q_{1}=N\left(\theta_{k}^{\left(i-1\right)},0.1^{2}\right).$$

For *i* > 2*n*_*P*_ (and *i* ≤ *n*_*S*_), we propose $\theta_{k}^{(i)c}$ from distribution *Q*_2_ defined by

$$Q_{2}=(1-\beta )N\left(\theta_{k}^{\left(i-1\right)},2.38^{2}\Sigma_{i}\right)+\beta N\left(\theta_{k}^{\left(i-1\right)},0.1^{2}\right),$$

where *β* = 0.05, as proposed by Roberts and Rosenthal (2009), and Σ_*i*_ is the empirical covariance of the parameter *θ*_*k*_ from the previous *i* iterations.

For *i* > *n*_*S*_, we use a grouped adaptive random walk Metropolis‐within‐Gibbs scheme, updating quartets $\theta_{G_{k}}^{\left(i\right)}=(\alpha_{k}^{\left(i\right)},\beta_{k}^{\left(i\right)},\mu_{k}^{\left(i\right)},\sigma_{k}^{\left(i\right)})$ jointly, before updating *ρ* independently as before. If *i* ≤ *n*_*S*_ + 2*n*_*P*_ and the quartet state is $\theta_{G_{k}}^{\left(i\right)}$, we propose candidates $\theta_{G_{k}}^{(i)c}$ from distribution

$$Q_{3}=\text{MVN}\left(\theta_{G_{k}}^{\left(i-1\right)},(0.1^{2})/4\right).$$

If *i* > *n*_*S*_ + 2*n*_*P*_, we propose $\theta_{G_{k}}^{(i)c}$ from distribution

$$Q_{4}=(1-\beta )\text{MVN}\left(\theta_{G_{k}}^{\left(i-1\right)},2.38^{2}\Sigma_{i}\right)+\beta \text{MVN}\left(\theta_{G_{k}}^{\left(i-1\right)},0.1^{2}/4\right),$$

where, again, *β* = 0.05 and Σ_*i*_ is the empirical variance–covariance matrix of the parameters $\theta_{G_{k}}^{\left(i\right)}$ from the previous *i* iterations. Finally, we update *ρ*.

Throughout, a candidate state is accepted using the standard Metropolis‐Hastings acceptance criterion. Because prior distributions for parameters are uniform and proposals are symmetric, this is effectively just a likelihood ratio. That is, we accept the candidate state with probability $\min(1,L^{c}/L)$, where *L* and *L* ^*c*^ are the likelihoods evaluated at the current and candidate states, respectively, with candidates lying outside their prior domains rejected. The MCMC procedure is summarised in Algorithm 1.
