# Supplementary material for: On spatial conditional extremes for ocean storm severity
Source: Environmetrics. 2019 Feb 26;30(6):e2562. doi: 10.1002/env.2562 (PMC6813651; doi:10.1002/env.2562)
Supplement: Supplementary file 1 — ENV_2562‐Supp‐0001‐Online supporting information.pdf [file ENV-30-na-s001.pdf]

Supplementary Material

On spatial conditional extremes for ocean storm severity

R. Shooter, E. Ross, J. Tawn and P. Jonathan

January 21, 2019

This document contains two plots to illustrate MCMC convergence for the NNS:N-S case; see Section 5 of the main document for details of this.

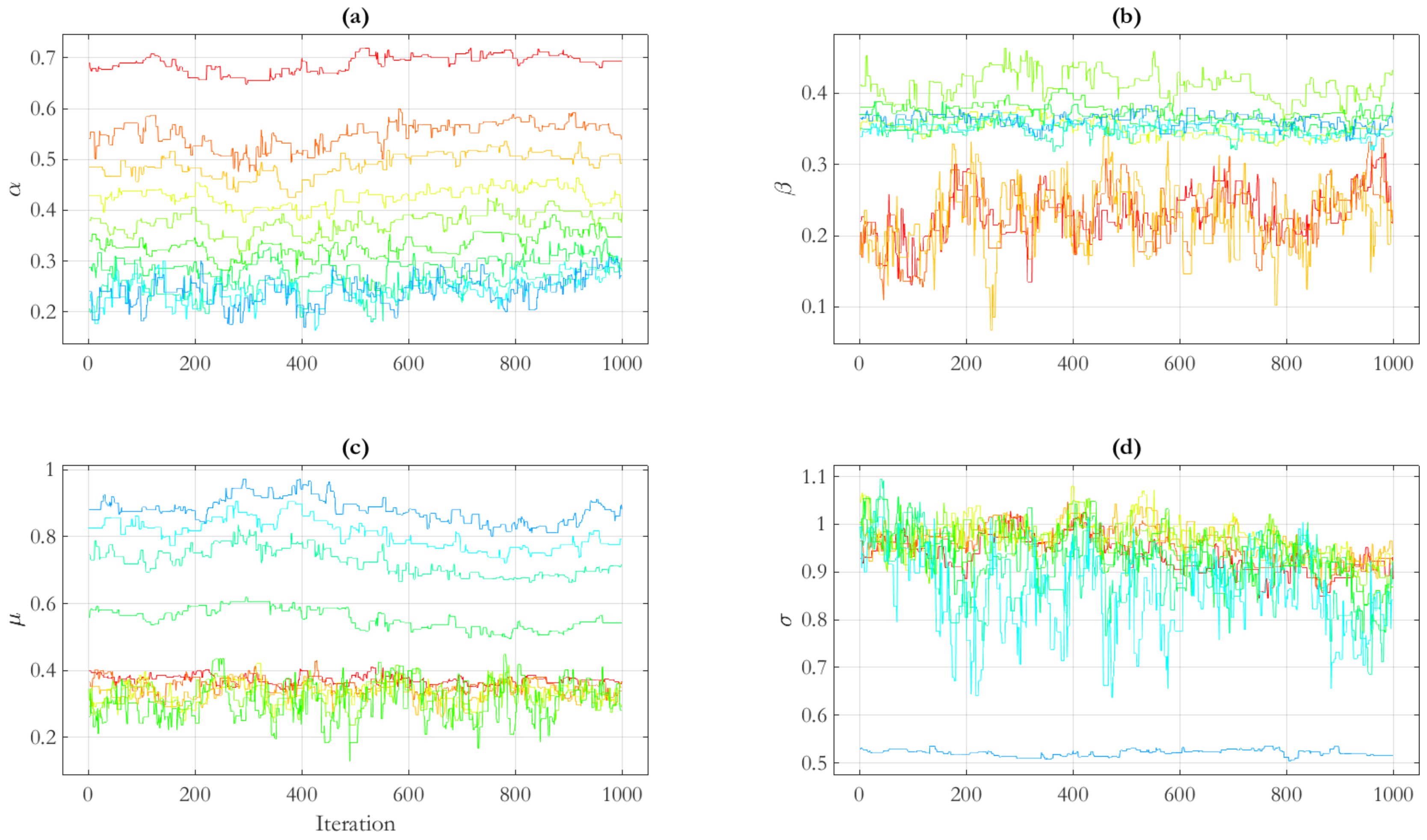

Figure 1: Trace plots of final 1000 iterations for parameters (a) α, (b) β, (c) μ and (d) σ from the fitted SCE model for the NNS:N-S application. Differently coloured lines represent different inter-location distances, as outlined in the main manuscript.

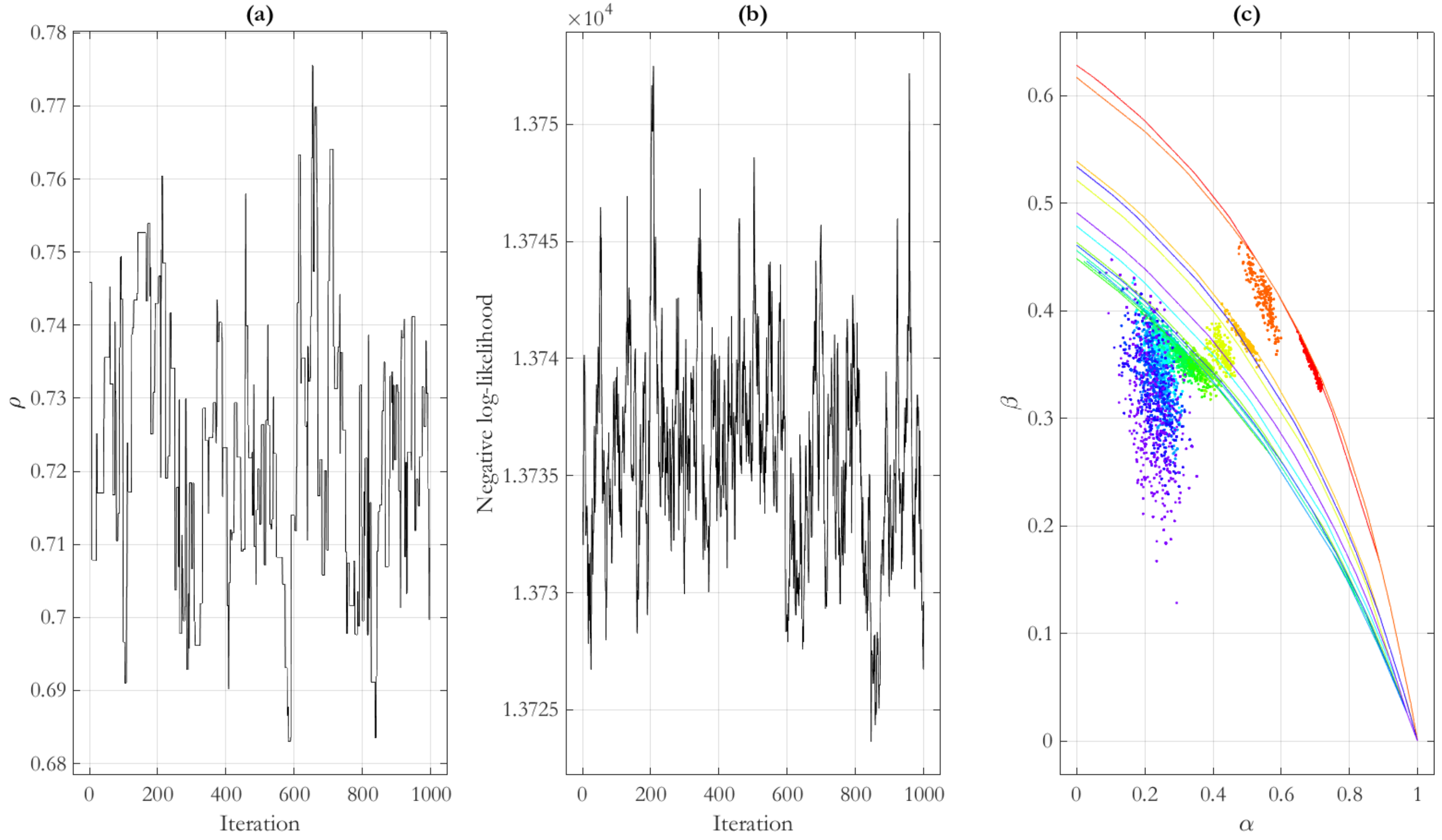

Figure 2: (a) Trace plots of final 1000 iterations for the parameter ρ from the fitted SCE model for the NNS:N-S application; (b) the final 1000 values of the negative log-likelihood for the SCE model; (c) Illustration of constraint boundaries from Keef et al. (2013), and estimates of (α, β) in relation to these. Differently coloured lines represent different inter-location distances, as outlined in the main manuscript.
